# Establishment of a dynamic nomogram including thyroid function for predicting the prognosis of acute ischemic stroke with standardized treatment

**DOI:** 10.3389/fneur.2023.1139446

**Published:** 2023-06-15

**Authors:** Yi Jiang, Chunhui Xie, Guanghui Zhang, Mengqian Liu, Yiwen Xu, Wen Zhong, Zhonglin Ge, Zhonghai Tao, Mingyue Qian, Chen Gong, Xiaozhu Shen

**Affiliations:** ^1^Department of Geriatrics, Bengbu Medical College Clinical College of Lianyungang Second People's Hospital, Lianyungang, China; ^2^Department of Neurology, The Affiliated Lianyungang Hospital of Xuzhou Medical University, Lianyungang, China; ^3^Department of Geriatrics, Lianyungang Second People's Hospital Affiliated to Jiangsu University, Lianyungang, China; ^4^Department of Neurology, Lianyungang Second People's Hospital, Lianyungang, China

**Keywords:** acute ischemic stroke, thyroid hormones, dynamic nomogram, predictive model, standardized treatment

## Abstract

**Purpose:**

Many patients with acute ischemic stroke (AIS) cannot undergo thrombolysis or thrombectomy because they have missed the time window or do not meet the treatment criteria. In addition, there is a lack of an available tool to predict the prognosis of patients with standardized treatment. This study aimed to develop a dynamic nomogram to predict the 3-month poor outcomes in patients with AIS.

**Methods:**

This was a retrospective multicenter study. We collected the clinical data of patients with AIS who underwent standardized treatment at the First People's Hospital of Lianyungang from 1 October 2019 to 31 December 2021 and at the Second People's Hospital of Lianyungang from 1 January 2022 to 17 July 2022. Baseline demographic, clinical, and laboratory information of patients were recorded. The outcome was the 3-month modified Rankin Scale (mRS) score. The least absolute shrinkage and selection operator regression were used to select the optimal predictive factors. Multiple logistic regression was performed to establish the nomogram. A decision curve analysis (DCA) was applied to assess the clinical benefit of the nomogram. The calibration and discrimination properties of the nomogram were validated by calibration plots and the concordance index.

**Results:**

A total of 823 eligible patients were enrolled. The final model included gender (male; OR 0.555; 95% CI, 0.378–0.813), systolic blood pressure (SBP; OR 1.006; 95% CI, 0.996–1.016), free triiodothyronine (FT3; OR 0.841; 95% CI, 0.629–1.124), National Institutes of Health stroke scale (NIHSS; OR 18.074; 95% CI, 12.264–27.054), Trial of Org 10172 in Acute Stroke Treatment (TOAST; cardioembolic (OR 0.736; 95% CI, 0.396–1.36); and other subtypes (OR 0.398; 95% CI, 0.257–0.609). The nomogram showed good calibration and discrimination (C-index, 0.858; 95% CI, 0.830–0.886). DCA confirmed the clinical usefulness of the model. The dynamic nomogram can be obtained at the website: predict model (90-day prognosis of AIS patients).

**Conclusion:**

We established a dynamic nomogram based on gender, SBP, FT3, NIHSS, and TOAST, which calculated the probability of 90-day poor prognosis in AIS patients with standardized treatment.

## Introduction

In 2019, the Global Burden of Disease study showed stroke was the second leading cause of death worldwide (1). In the same year, the literature showed that stroke was also the leading cause of death in China (2). In the face of such a heavy disease burden, any assistance provided to clinicians is beneficial. Early prediction of a patient's prognosis can help clinicians choose appropriate treatment options and communicate effectively with patients and their families, thereby improving their quality of life.

Treatment during an AIS attack is closely related to the prognosis. Thrombolysis is currently an important treatment for stroke and provides significant clinical benefits to patients (3, 4), but many patients are excluded because of difficulties in meeting the indications for thrombolysis or embolization. The strict time-window for treatment remained a major limitation (5). A study indicated that 69% of people missed treatment due to delayed admission to the hospital (6). Meanwhile, milder stroke symptoms (NIHSS <6) were also one of the major reasons why patients missed thrombolytic therapy (7).

The prognosis of stroke may be influenced by a variety of factors (8–10). In recent years, many studies have pointed to an association between thyroid hormones and the prognosis of stroke. A study including 702 patients showed that low free triiodothyronine (FT3) levels were independently associated with poor functional outcomes and mortality 3 months after stroke onset (11). A study included 199 patients with AIS identified low thyroid stimulating hormone (TSH) levels as an independent risk factor for adverse outcomes at 3 months (12). Another study that included 848 AIS patients with intracranial atherosclerotic stenosis showed that high free thyroxine (FT4) levels were associated with poor prognosis (13).

These results suggest that we need to consider the possible influence of thyroid hormone levels on stroke when predicting stroke prognosis.

Nomograms are widely used as a tool for visualizing predictive models. However, there is a lack of an available tool to help clinicians predict the prognosis of populations receiving standardized treatment for AIS. This study aimed to develop a predictive model for predicting the severity at discharge of AIS patients with standardized treatment and to develop a tool easily used by clinical staff.

## Methods

### Study design and population

This was a retrospective study. We collected the clinical data of patients with AIS who underwent standardized treatment at the First People's Hospital of Lianyungang between 1 October 2019 and 31 December 2021 and at the Second People's Hospital of Lianyungang between 1 January 2022 and 17 July 2022.

Standardized treatment was given to patients according to guidelines (5) for respiratory support, blood pressure, temperature and glucose management, and antiplatelet therapy but did not include thrombolysis and endovascular therapy.

Patients were enrolled in this study if they met the following criteria: (1) age ≥ 18 years old; (2) onset within 48 h; (3) presence of acute ischemic lesions in anterior circulation, which were confirmed by imaging methods (magnetic resonance imaging or computed tomography); and (4) received standardized treatment. The exclusion criteria for this study were (1) intracranial hemorrhage or mass lesion; (2) with severe infection or septic shock; (3) liver or renal failure; (4) received thrombolysis or mechanical thrombectomy; and (5) incomplete laboratory, clinical, or follow-up data.

Ethical approval for this study was obtained from the ethics committees of Lianyungang First People's Hospital (No. KY-20210917001-01).

### Define

Baseline clinical information of all enrolled patients was collected from the database, including the following data: age, gender, systolic blood pressure (SBP), diastolic blood pressure (DBP), history of hypertension (HTN), diabetes (DM), atrial fibrillation (AF), aspirin used, coronary heart disease(CHD), current smoking (any usage of cigarettes per day in the past 30 days).

Laboratory findings at admission included TSH, FT4, FT3, blood glucose (Glu), glycated hemoglobin (HbA1c), total cholesterol (TC), low-density lipoprotein cholesterol (LDL), and homocysteine (Hcy). All laboratory indicators and blood samples were taken within 24 h of admission.

According to the Trial of Org 10172 in Acute Stroke Treatment (TOAST) classification, acute ischemic stroke was divided into large-artery atherosclerosis (LAA), cardioembolic (CE), small artery occlusion (SAO), stroke of other determined etiology (SOE), and stroke of undetermined etiology(SUE) (14). SAO, SOE, and SUE subtypes were combined into one group and defined as the “other” subgroup.

The National Institute of Health stroke scale (NIHSS) consists of 12 categories with a total score of 42 and is used to assess neurological deficits (15). Patients were assessed for stroke severity by NIHSS score, with ≤ 4 being mild stroke and >4 being severe stroke (16).

The modified Rankin Scale (mRS) is often used to assess the AIS degree of disability and as an outcome variable to assess recovery from stroke in the study about AIS (17, 18). It could be found in the Supplementary material. The mRS at 90 days was the outcome variable in this study. Poor prognosis was defined as an mRS of >2, and excellent prognosis was defined as an mRS of ≤ 2 (19). A professional neurologist at our stroke center used the telephone review to evaluate mRS scores, who was blinded to baseline clinical and imaging information.

### Candidate predictors

The candidate predictors include age, gender, SBP, DBP, smoking, HTN, AF, CHD, aspirin, HbA1c, TC, LDL, Hcy, TSH, FT3, FT4, NIHSS, and TOAST.

### Statistical analysis

The analyses were done with R version 4.2.2 (R Core Team 2022) and some statistical analyses based on additional packages (“glmnet,” “rms,” “dcurves,” and “shinyPredict”). Statistical tests were two sided and a p-value of < 0.05 was considered to be statistically significant. The Kolmogorov–Smirnov test was used to test the normality of the data. For parameters with continuous data, the normal distribution was expressed as mean ± standard deviation, and the skewed distribution was all expressed as median and quartile range (P25–P75). Count data were expressed as rate (%). According to 90-day mRS Scores, all patients were divided into two groups: the poor prognosis group (mRS > 2) and the good prognosis group (mRS ≤ 2). Normal distribution data were analyzed by independent sample *t*-test, and the non-normal distribution data were analyzed by Mann–Whitney *U*-test. Fisher's exact test or the chi-square test was used to compare categorical variables as appropriate.

The LAA group in TOAST was defined as a dummy variable in the regression since TOAST was a multicategorical variable. The least absolute shrinkage and selection operator (LASSO) method was used to screen the final predictors, and λ was adjusted appropriately to simplify the model to avoid overfitting of the model (the value of λ is between λ that minimizes the model error and one of its standard deviations).

The bootstrap method was used for internal verification, and the number of resampling was 100. The C-index and the receiver operating characteristic curve (ROC) were used to evaluate the accuracy of the model in predicting outcomes. The calibration curves were used to evaluate the fitting degree of the model. The decision curve analysis (DCA) was used to demonstrate the net benefit at an arbitrary probability threshold to evaluate the clinical usefulness of the model (20).

The model was developed by multiple logistic regression and visualized by drawing a nomogram. By scoring each variable in the nomogram and summing them, the total score points to the vertically corresponding predicted probability. A dynamic nomogram was produced to simplify the use. Click on the groups and enter values to display the predicted probabilities on the dynamic nomogram.

## Results

### Baseline characteristics of study participants

**Figure 1:**
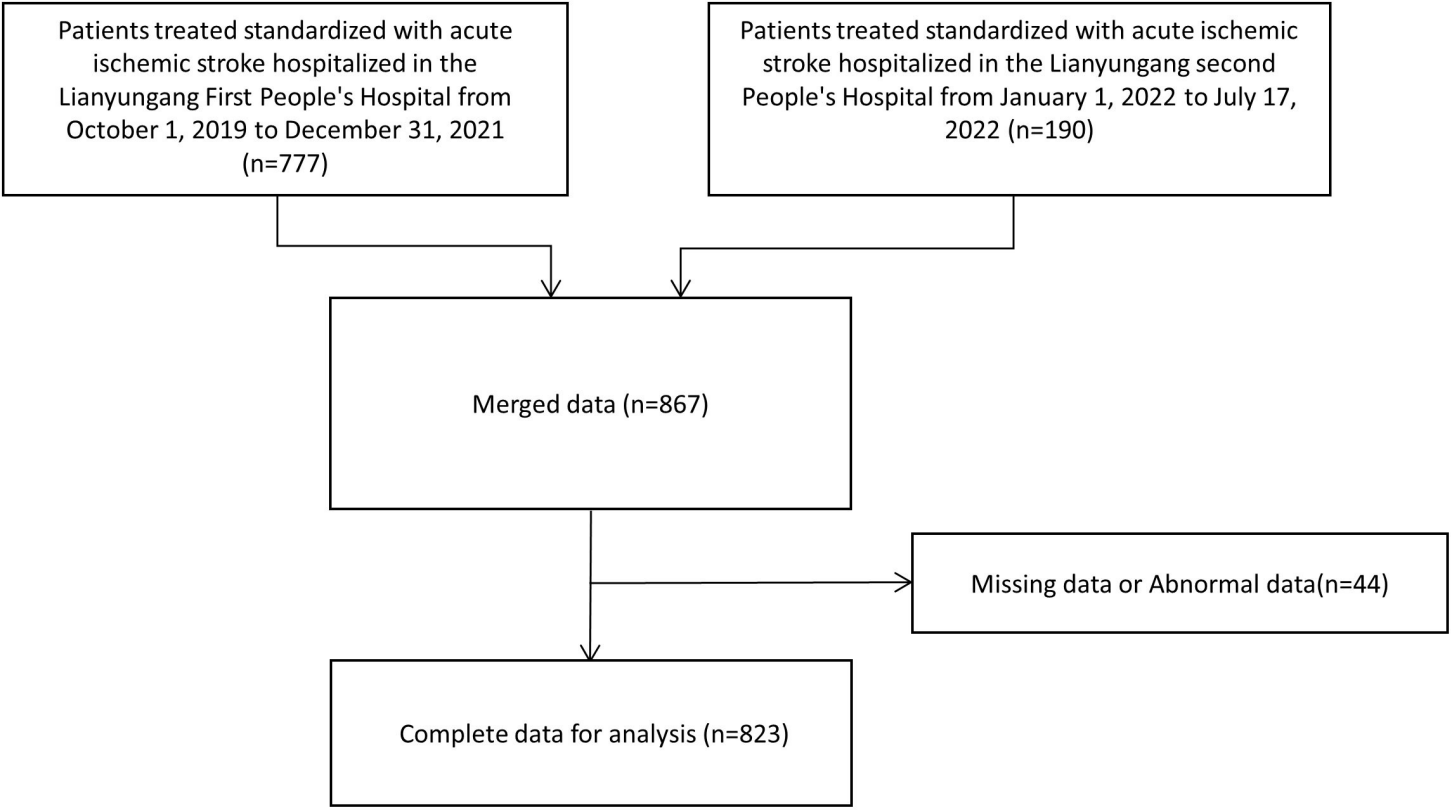
Subjects screening flowchart.

The study population comprised 777 patients from Lianyungang First People's Hospital and 190 patients from Lianyungang Second People's Hospital. After excluding missing values and abnormal data, 823 patients were finally included in this study (Figure 1). According to the baseline data, age, gender, SBP, AF, Glu, TSH, FT4, and TOAST showed statistical differences between the two groups. The poor prognosis group had older age, more females, DM, higher SBP, higher FT4, and lower Glu (Table 1).

**Table 1:** Baseline characteristics.

	**Total (*n* = 823)**	**Excellent prognosis (mRS ≤ 2, *n* = 571)**	**Poor prognosis (mRS > 2, n = 252)**	***p***
**Parameter**				
Male-no. (%)	474 (57.6)	356 (62.3)	118 (46.8)	< 0.001
Age, Median (IQR)-years	67.00 [58.00, 75.00]	66.00 [57.50, 73.00]	69.00 [61.75, 78.00]	< 0.001
SBP, Median (IQR)-mmHg	148.00 [138.00, 158.00]	147.00 [138.00, 156.00]	150.00 [139.75, 160.00]	0.027
DBP, Median (IQR)-mmHg	86.00 [79.00, 96.00]	85.00 [78.50, 96.00]	87.00 [79.00, 97.00]	0.612
**Medical history**				
Smoking-no. (%)	241 (29.3)	171 (29.9)	70 (27.8)	0.584
Drinking-no. (%)	199 (24.2)	141 (24.7)	58 (23.0)	0.667
Diabetes mellitus-no. (%)	223 (27.1)	150 (26.3)	73 (29.0)	0.473
Hypertension-no. (%)	558 (67.8)	387 (67.8)	171 (67.9)	1.000
Atrial fibrillation-no. (%)	81 (9.8)	44 (7.7)	37 (14.7)	0.003
Chronic heart disease-no. (%)	84 (10.2)	53 (9.3)	31 (12.3)	0.232
Aspirin-no. (%)	151 (18.3)	103 (18.0)	48 (19.0)	0.805
**Biochemical**				
Glu, Median (IQR)-mmol/L	5.48 [4.80, 7.09]	5.37 [4.71, 6.87]	5.82 [5.07, 7.46]	< 0.001
HbA1c, Median (IQR)-mmol/L	6.00 [5.60, 7.30]	6.00 [5.60, 7.20]	6.10 [5.60, 7.50]	0.309
TC, Median (IQR)-mmol/L	4.50 [3.77, 5.22]	4.51 [3.81, 5.22]	4.46 [3.68, 5.19]	0.417
LDL, Median (IQR)-mmol/L	2.53 [2.03, 3.02]	2.53 [2.04, 2.99]	2.55 [2.01, 3.13]	0.430
Hcy, Median (IQR)-umol/L	9.80 [8.00, 12.60]	9.70 [8.10, 12.60]	9.85 [7.80, 12.60]	0.901
TSH, Median (IQR)-mmol/L	1.57 [0.96, 2.40]	1.66 [1.06, 2.51]	1.29 [0.73, 2.17]	< 0.001
FT3, Mean (SD)-mmol/L	4.64 ± 0.66	4.67 ± 0.62	4.57 ± 0.73	0.049
FT4, Median (IQR)-mmol/L	12.12 [10.95, 13.48]	12.05 [10.92, 13.30]	12.41 [11.08, 13.91]	0.016
**NIHSS** **>-no. (%)**	240 (29.2)	62 (10.9)	178 (70.6)	< 0.001
**TOAST-no. (%)**				< 0.001
LAA	418 (50.8)	257 (45.0)	161 (63.9)	
CE	83 (10.1)	44 (7.7)	39 (15.5)	
Other	322 (39.1)	270 (47.3)	52 (20.6)	

SD, standard deviation; IQR, interquartile range; SBP, systolic blood pressure; DBP, diastolic blood pressure; Glu, blood glucose; HbA1c, glycated hemoglobin; TC, total cholesterol; LDL, low-density lipoprotein cholesterol; Hcy, homocysteiney; TSH, thyroid stimulating hormone; FT3, free triiodothyronine; FT4, free thyroxine; NIHSS, National Institute of Health stroke scale; According to the TOAST, Trial of Org 10172 in Acute Stroke Treatment; LAA, large-artery atherosclerosis; CE, cardioembolic; SAO, small artery occlusion; SUE, stroke of other determined etiology; SOE, stroke of undetermined etiology. The other subgroup was defined as SAO, SOE, and SUE subtypes merged.

**Figure 2:**
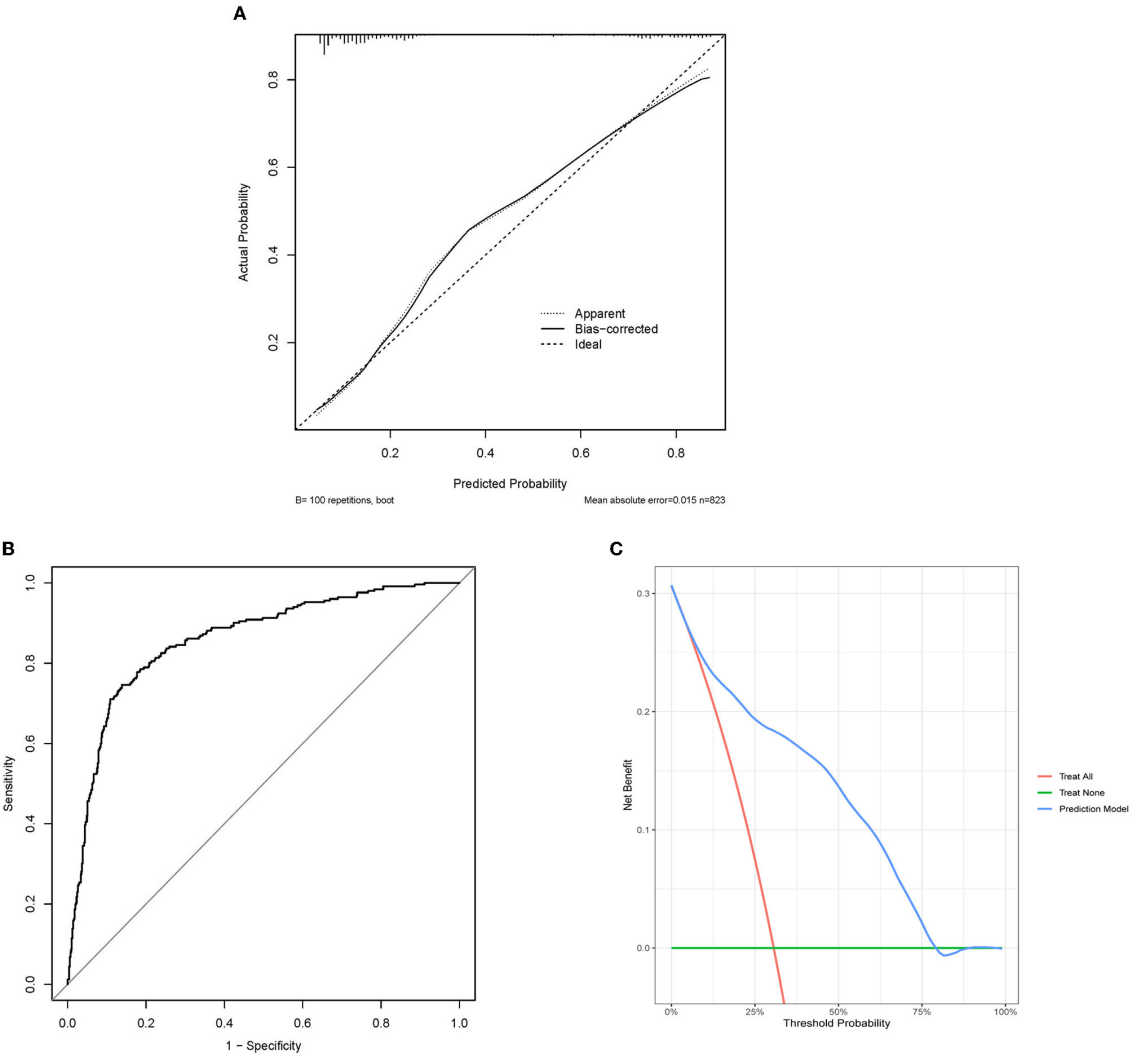
Evaluation of predictive models. **(A)** Calibration plot of internal verification. 100 repetitions bootstrap, mean absolute error is 0.015. The diagonal line in the plot represents an ideal model. The dotted line represents the predictive capabilities of the current model, whereas the solid line corrects for bias in the model. **(B)** The receiver operating characteristic curve about the final model. The area under the curve is 0.858, and 95% Confidence interval is 0.830–0.886. **(C)** DCA of the nomogram. DCA might explain the clinical benefit, assuming a probability threshold of 60% agreed upon by physicians and patients, and a net gain of 0.1 approximated in the figure, which could be interpreted as using the model to select interventions for 100 patients, with 10 additional patients of patients (true positives) being correctly intervened without paying a loss (false positives). ROC, the receiver operating characteristic curve; DCA, the decision curve analysis.

**Figure 3:**
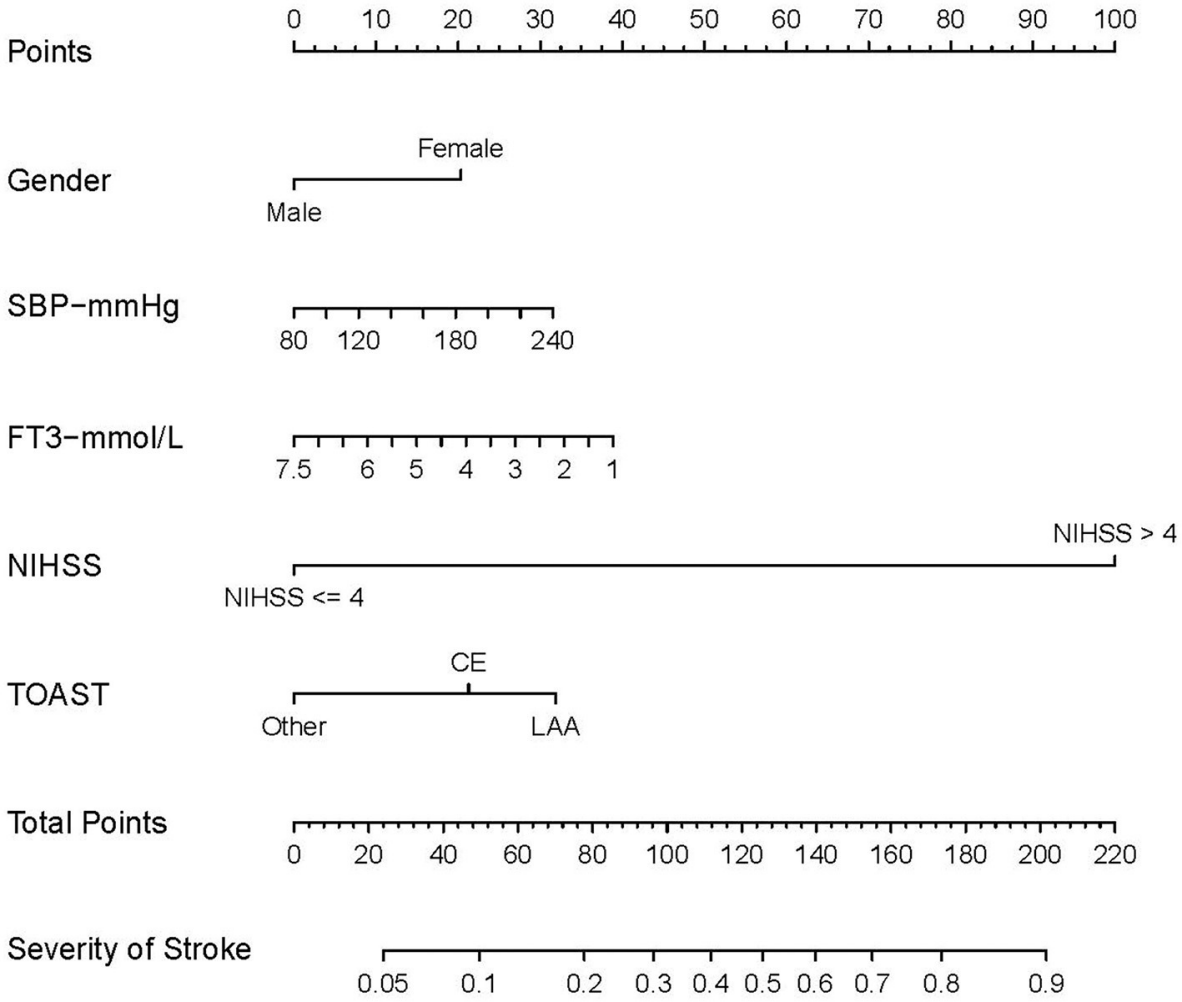
Nomogram for predicting prognosis of AIS. The nomogram includes gender, SBP, FT3, NIHSS, and TOAST. The nomogram used to predict the risk of poor prognosis in AIS patients and its algorithm are as follows. First, the corresponding score for the patient with AIS is found on the score line at the top of each variable; then all the scores are summed to find the corresponding score on the total score. Finally, the predicted probability corresponding to the patient is found on the predicted value line. SBP, systolic blood pressure; FT3, free triiodothyronine; NIHSS, National Institute of Health stroke scale. According to the TOAST, Trial of Org 10172 in Acute Stroke Treatment; LAA, large-artery atherosclerosis; CE, cardioembolic; SAO, small artery occlusion; SUE, stroke of other determined etiology; SOE, stroke of undetermined etiology. The other subgroup was defined as SAO, SOE, and SUE subtypes merged.

LASSO was used to screen the final predictors (Figures 2, 3). The process of variable screening was shown in the Supplementary material. The final model included gender (OR 0.555; 95% CI, 0.378–0.813), SBP (OR 1.006; 95% CI, 0.996–1.016), CE (OR 0.736; 95% CI, 0.396–1.36), other (OR 0.398; 95% CI, 0.257–0.609), FT3 (OR 0.841; 95% CI, 0.629–1.124), and NIHSS (OR 18.074; 95% CI, 12.264–27.054).

### Model verification

The C-index of the final model was 0.858, and 95% CI was 0.830–0.886 (Figure 2A). The bootstrap method corrected C-index was 0.851. The calibration curves showed good fit, which confirmed the accuracy of our model in predicting prognosis (Figure 2B). The DCA curves showed good performance, which implied that patients would achieve clinical benefits using our model (Figure 2C).

### Establishment of nomogram

**Figure 4:**
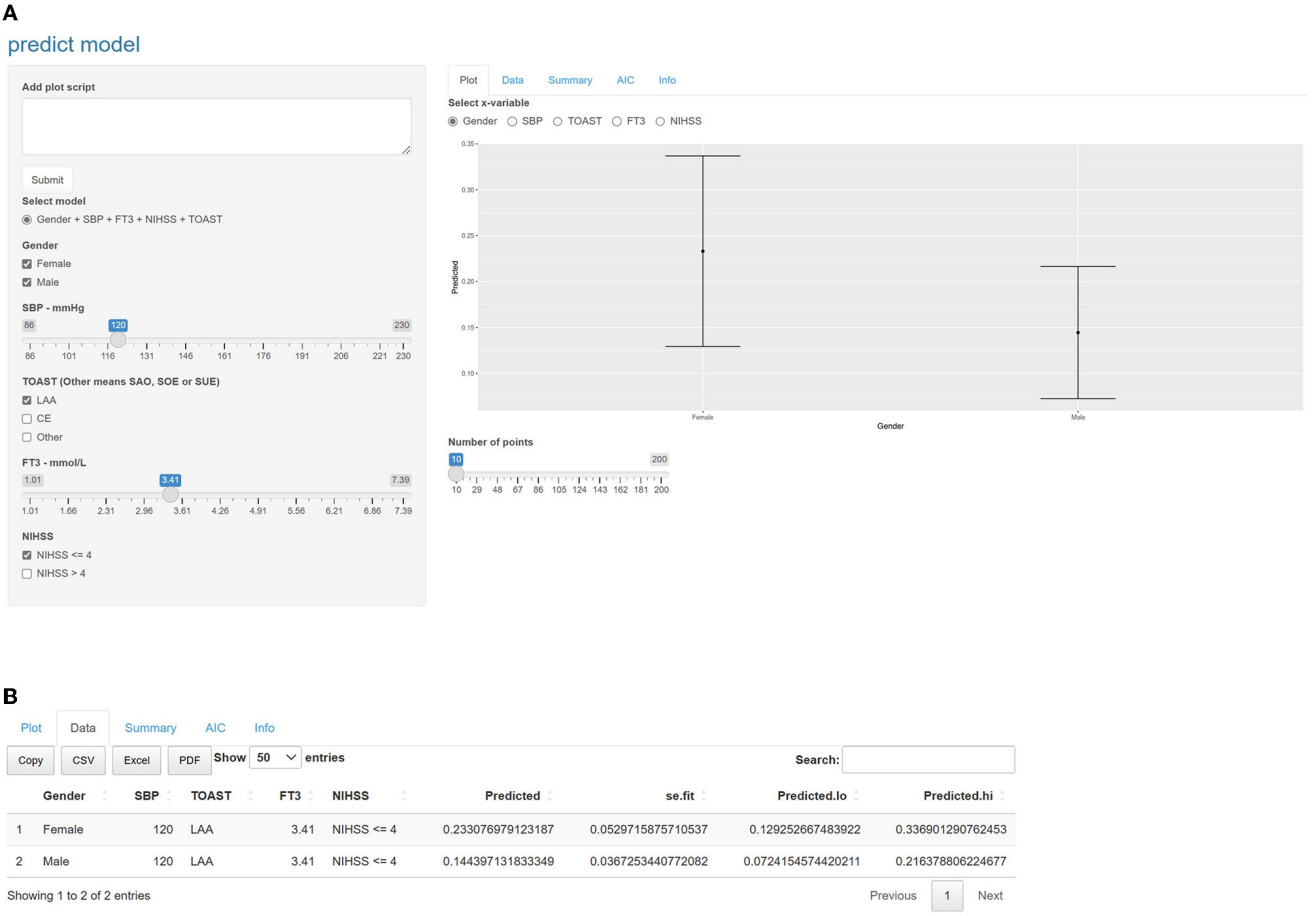
Intuitive interface of the online dynamic nomogram. Users can input the values of the variables and a graph of the probability of poor prognosis at 3 months is automatically plotted on the right side of the interface, or click on the data option to get accurate predictive value. **(A)** Selecting female, male, SBP = 120mmHg, LAA, FT3 = 3.41mmol/L and NIHSS ≤ 4, selecting the gender grouping on the right side of the interface, you would see the prediction graphs representing female and male respectively. The length of the line indicates the 95% confidence interval, and the middle dot indicates the predicted probability. **(B)** Clicking on the data option, you can see that the predicted value for female was 0.233 and for male was 0.144. We provide the corresponding network address: predict model (90-day prognosis of AIS patients). SBP, systolic blood pressure; FT3, free triiodothyronine; NIHSS, National Institute of Health stroke scale. According to the TOAST, Trial of Org 10172 in Acute Stroke Treatment; LAA, large-artery atherosclerosis; CE, cardioembolic; SAO, small artery occlusion; SUE, stroke of other determined etiology; SOE, stroke of undetermined etiology. The other subgroup was defined as SAO, SOE, and SUE subtypes merged.

A nomogram was created based on the five significant predictors in the final model (Figure 3). The scores corresponding to each variable were added to calculate the total score and estimate the probability of poor prognosis. In addition, we constructed a dynamic nomogram to provide an intuitive web interface with the direct input of variables to obtain the predicted probabilities of AIS for ease of use (Figure 4). The corresponding network address is as follows: predict model (90-day prognosis of AIS patients).

## Discussion

This study population was limited to acute ischemic stroke with standardized treatment. A dynamic nomogram was established based on five variables including gender, SBP, FT3, NIHSS, and TOAST. The predictive performance of this model was satisfactory (C-index was 0.858). The variables in the nomogram were indicated by previous studies for correlation with the prognosis of stroke. This indicated that the model was plausible rather than being fitted based on the data without consideration.

Two reviews from different periods showed that women had a higher risk of ischemic stroke than men and may have a worse functional prognosis (21, 22). The relationship between the NIHSS score and stroke prognosis has been well established and widely used to assess the severity of the stroke and to predict poor prognosis (23, 24). The prognosis of stroke in patients with hypertensive disorders is usually poor. Patients with hypertension have less penumbral tissue and larger infarcts than patients with normal blood pressure although lower blood pressure is also detrimental in stroke (25). CE has a more severe presentation and worse prognosis than other subtypes of stroke (26). The nomogram showed a greater risk of LAA, which seemed inconsistent with the current findings. We considered the study population included fewer patients with CE, and the milder degree of stroke was also a reason for patients to take standardized treatment. Due to the higher severity of CE, patients may have received thrombolytic or interventional therapy and were excluded from the study.

Numerous studies have indicated the association between thyroid hormones and ischemic stroke. Low FT3 values were reported to be independently associated with poor functional outcomes and mortality at 3 months after stroke onset in 702 consecutive acute stroke patients (11). A retrospective case–control study in patients with early-onset ischemic stroke without diabetes and hypertension revealed significantly lower levels of FT3 and FT4 in patients with ischemic stroke than in the controls (27). In another investigation, patients with poor outcomes had significantly lower FT3 and serum total triiodothyronine levels and FT3/FT4 ratio but higher FT4 levels (28). A lower TSH level was associated with unfavorable functional outcomes in acute ischemic patients after endovascular therapy (12). In this study, FT3 was included in the model, and FT4 and TSH were excluded in the screening variables process. The probable explanation was that FT3 had more weight in the model than FT4 and TSH. The pathophysiological mechanisms of thyroid hormones in stroke were still unclear. It has been suggested that thyroid hormones may play a role in neurological recovery after stroke, such as neuronal plasticity, neurogenesis, and angiogenesis (29).

DCA was proposed in 2006 and was widely used for the evaluation (20) of predictive models. Flexible selection of the probability threshold interval is a key step in the evaluation of clinical benefit (30). Our model showed clinical benefits below a probability threshold (roughly between 5 and 80%) even though we would perhaps not use such a large probability threshold.

This study had some limitations: (1) This was a retrospective study, with inherent limitations associated with retrospective analyses; (2) The sample size was relatively small and the data collected might lack some potential predictors, which might decrease the predictive power; (3) The sample was taken from two hospitals nearby, so we combined the data into a single dataset to improve model stability and avoid bias from outlier validation. Hence, external validation of the model at other stroke centers is required; (4) In this study, the sample size of the SUE and SOE subgroups was small, and they were combined with the SAO subgroup, so there should be more caution in the interpretation of the results about the TOAST 5. The analysis of fatal cases (mRS = 6) was lacking because of the small number of cases with fatal outcomes (*n* = 2). Despite these limitations, to the best of our knowledge, this is the first study to include thyroid hormones as predictors in a nomogram model to predict the 90-day prognosis of AIS patients with standardized treatment.

## Conclusion

This study established a dynamic nomogram comprising gender, SBP, FT3, NIHSS, and TOAST to predict the 90-day prognosis of AIS patients with standardized treatment. It provided an accurate and convenient tool for clinicians to predict the 90-day prognosis of AIS patients with standardized treatment.

## Data availability statement

The raw data supporting the conclusions of this article will be made available by the authors, without undue reservation.

## Ethics statement

The studies involving human participants were reviewed and approved by the Ethics Committee of Lianyungang First People's Hospital (No. KY-20210917001-01). The patients/participants provided their written informed consent to participate in this study.

## Author contributions

GZ and XS conceived and designed the research. YJ and CX analyzed the data and drafted the manuscript. YX and ML collected the data and performed the research. All authors reviewed and edited the manuscript and approved the final version of the manuscript.
