# Supplementary material for: Establishment of a dynamic nomogram including thyroid function for predicting the prognosis of acute ischemic stroke with standardized treatment
Source: Front Neurol. 2023 Jun 15;14:1139446. doi: 10.3389/fneur.2023.1139446 (PMC10311209; doi:10.3389/fneur.2023.1139446)
Supplement: Supplementary file 1 [file Data_Sheet_1.docx]

1. The modified Rankin Scale (mRS) scores

| The modified Rankin Scale (mRS) scores | |
| --- | --- |
| 0 | No symptoms at all |
| 1 | No significant disability: despite symptoms, able to carry out all usual duties and activities |
| 2 | Slight disability: unable to perform all previous activities but able to look after own affairs without assistance |
| 3 | Moderate disability: requiring some help but able to walk without assistance |
| 4 | Moderately severe disability: unable to walk without assistance and unable to attend to own bodily needs without assistance |
| 5 | Severe disability: bedridden, incontinent and requiring constant nursing care and attention |
| 6 | Death (Attributed to stroke, or other stroke complications) |

1. Lasso regression for screening variables

LASSO was used to screen the final predictors (FIG.1). The [minimum](javascript:;) value of λ (minλ) represented the model with the lowest error was 0.0054 (Logarithmically converted to -5.2223), when choose minλ, the number of variables selection in final model was 14. For simplified the final model, we choose was λ value = 0.01381 (Logarithmically converted to -4.28236). The maximum λ (maxλ) is equal to the minλ plus a standard deviation was 0.07496 (Logarithmically converted to -2.59087). The final λ lies between the values of minλ and maxλ, the method of fitting the model was feasible (FIG.2).


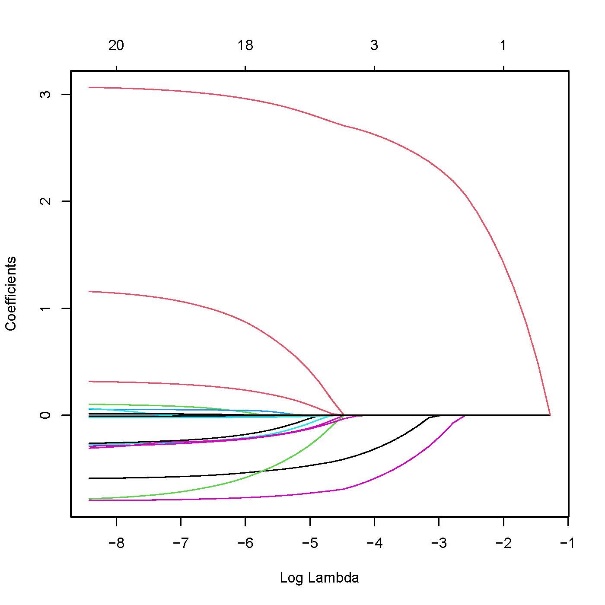


FIG.1 LASSO coefficient profiles of potential predictors. The y-aixs indicate the regression coefficients of predictors, the x-aixs indicate Log-transformed λ-value As the value of λ increases, the regression coefficients for all potential predictions included in the model converge to zero.


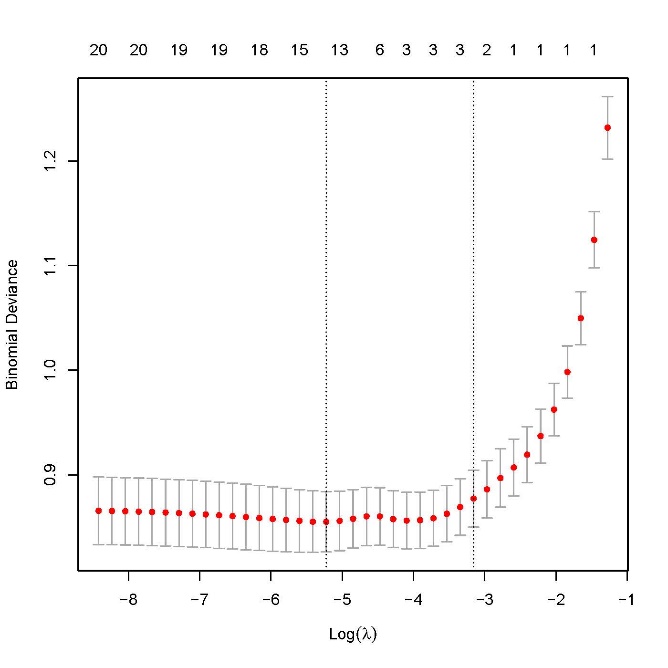


FIG.2 Screening of the optimal penalization coefficient in the LASSO regression. The dotted line on the left is the Log-transformed λ-value that gives minimum mean cross-validated error, and the dotted line on the right represents Log-transformed λ-value of the most regularized model such that the cross-validated error is within one standard error of the minimum Log-transformed λ-value.
